# Painless Miscarriage

**Published:** 1868-07-15

**Authors:** 


***Painless Miscarriage.***

Oct. 10th, 1867, was called to Mrs. D., pregnant about five months. While engaged in her ordinary household duties, without the least premonition, there was rupture of the membranes, and discharge of the amniotic fluid.

11*th*. Ordered *Tinct. ergot* in drachm doses every hour for six hours, but without effect. In the evening I found the os uteri well dilated, right shoulder presenting, with protrusion of the hand and arm, though she had not felt a single pain, but only a weight, or dragging sensation, when she stood upon her feet.

Not thinking it necessary to turn, as the fœtus was small, I introduced a finger on each side of the neck, and brought down the head; then with slight traction, and a bearing down effort on her part, it was easily delivered. There was also another fœtus, with breech presenting, which I delivered in like manner, by bringing down the feet.

After waiting an hour, I administered *Chloroform*, and removed the placenta, which was so firmly adherent that it was impossible to detach it except by taking it away in pieces.

The lady had a good getting up, and in ten days was able to perform her household duties, not having had a single pain from first to last.

I have since attended her in a miscarriage brought on in the same way, but she had a few pains.

Dr. Habershon, of London, in April and May last, treated a patient, now under our personal charge. Our patient preserved several of the original prescriptions, which are here inserted, as showing how they do these things in England. Their object is patent, and although simple enough in themselves, they differ in a marked degree from the formulæ which come from time to time from continental medical advisers:

℞. *Ferri arseniatis*, . gr. $\frac{1}{16}$;

*Zinci Valer.* . . gr. j.;

*Pil. Rhei comp.* . gr. j.;

*Extr. Hyoscy.* . . gr. j.;

M. *Ft. pil.* *Mitte* xvj. To be silvered. One to be taken every morning.

℞. *Potassii Bromidi* . ʒ ss.

*Ext. cinch. fl. liq.* . . ʒ i.

*Spir. ammon. arom.* . ʒ ij.

*Inf. aurantii ad.* . . ℥ vj.

M. A sixth part to be taken twice a day. S. O. H.

About three weeks subsequently, these formulæ were replaced by the following:

℞. *Acidi nitrici, dil.* . ʒ j.

*Acidi hydrochlor., dil.* . ʒ j.

*Quinæ dis.* . . . gr. vj.

*Ext. Taraxaci* . . ʒ ij.

*Syrupi aurantii* . . ʒ ij.

*Aquæ ad.* . . . ℥ vj.

M. A sixth part to be taken twice a day with water.

S. O. H.

Green Valley, Ill., May 1, 1868.

J. Adams Allen, M.D.—*Dear Sir*:—It becomes my painful duty to inform you of the death of O. S. Wood, M.D., a member of our class. He graduated in the class of 1859–60. He died at the residence of Mr. S. Woodrow, Green Valley, Ill., March 28th, 1868.

Very truly yours, Thos. C. Murphy, M.D.

